# Patient‐reported outcomes: active surveillance vs radical therapies in low‐risk prostate cancer

**DOI:** 10.1111/bju.70167

**Published:** 2026-02-16

**Authors:** Mulham Al‐Nader, Claudia Kesch, Osama Mahmoud, Boris A. Hadaschik, Jan Fichtner, Guenther Carl, Günter Feick, Martin Burchardt, Volker Zimmermanns, Lukas Prause, Bülent Polat, Andreas Blana, Marcus Horstmann, Matthias Saar, Petra Miglierini, Kinan Almansur, Lukas Hefermehl, Daniel Porres, Inga Peters, Kristina Wiens, Rein Jüri Palisaar, Alexander Winter, Andreas Neisius, Eva‐Maria Kunzmann, Nina Natascha Harke, Christian Bolenz, Mohamad Hatem Albarghouth, Lukas Manka, Mario Kramer, Ferdinand Luger, Thomas Knoll, Jesco Pfitzenmaier, Marko Brock, Julia Schittko, Jens Peter Sommer, Matthias Reichert, Sebastian Lenart, Philipp Huber, Sameh Hijazi, Anna Calderaro, Thomas Hermanns, Jens Tonhauser, Christoph Kowalski, Nora Tabea Sibert

**Affiliations:** ^1^ Department of Urology University Hospital Essen Essen Germany; ^2^ Department of Urology and Urologic Oncology Alfried Krupp Hospital Essen Germany; ^3^ Department of Urology Johanniter Hospital Oberhausen Germany; ^4^ Bundesverband Prostatakrebs Selbsthilfe e.V. Bonn Germany; ^5^ Department of Urology University Medicine Greifswald Greifswald Germany; ^6^ Department of Urology Siloah St. Trudpert Klinikum Pforzheim Germany; ^7^ Department of Urology Kantonsspital Aarau Aarau Switzerland; ^8^ Department of Radiation Oncology University Hospital Wuerzburg Wuerzburg Germany; ^9^ Department of Urology Fuerth Hospital Fuerth Germany; ^10^ Department of Urology Gütersloh Hospital Gütersloh Germany; ^11^ Department of Urology and Pediatric Urology University Hospital, RWTH Aachen University Aachen Germany; ^12^ Department of Radiation Oncology HFR Fribourg Fribourg Switzerland; ^13^ Department of Urology Kantonsspital Baden Baden Switzerland; ^14^ Department of Urology Leverkusen Hospital Leverkusen Germany; ^15^ Department of Urology Nordwest Hospital Frankfurt am Main Germany; ^16^ Department of Urology Fulda Hospital Fulda Germany; ^17^ Department of Urology Ruhr‐University Bochum Bochum Germany; ^18^ Marienhospital Herne Herne Germany; ^19^ University Hospital for Urology, Klinikum Oldenburg, School of Medicine and Health Sciences Carl von Ossietzky University Oldenburg Oldenburg Germany; ^20^ Department of Urology, Hospital of the Brothers of Mercy Trier Medical Campus University Mainz Trier Germany; ^21^ Department of Urology Christliches Klinikum Paderborn, Betriebsstätte: Brüderkrankenhaus St. Josef Paderborn Paderborn Germany; ^22^ Department of Urology Hannover Medical School Hannover Germany; ^23^ Department of Urology Ulm University Hospital Ulm Germany; ^24^ Department of Urology, Jena University Hospital Friedrich‐Schiller University Jena Germany; ^25^ Department of Urology Academic Hospital Braunschweig Braunschweig Germany; ^26^ Department of Urology Städtisches Klinikum Lüneburg Lüneburg Germany; ^27^ Ordensklinikum Linz, Barmherzige Schwestern Linz Linz Austria; ^28^ Department of Urology, Klinikum Sindelfingen‐Boeblingen University of Tuebingen Tuebingen Germany; ^29^ Department of Urology Evangelic Hospital Bethel Bielefeld Germany; ^30^ Department of Urology Stiftungsklinikum PROSELIS Recklinghausen Germany; ^31^ Clinic for Urology, Uro‐Oncology, Roboter‐Assisted and Focal Therapy University Hospital Magdeburg Magdeburg Germany; ^32^ Department of Urology Rems‐Murr‐Hospital Winnenden Germany; ^33^ Department of Urology University Medical Center Goettingen Goettingen Germany; ^34^ Department of Urology and Andrology St. John of God Hospital Vienna – Krankenhaus der Barmherzigen Brüder Wien Vienna Austria; ^35^ Urologie St. Anna Lucerne Switzerland; ^36^ Klinikum Ibbenbüren Ibbenbüren Germany; ^37^ Department of Urology Klinik am Eichert Göppingen Göppingen Germany; ^38^ Department of Urology Klinik Hirslanden and Klinik im Park Zürich Switzerland; ^39^ Department of Urology Hegau‐Bodensee‐Kliniken Singen Singen Germany; ^40^ Deutsche Krebsgesellschaft e. V. Berlin Germany; ^41^ Oncological Health Services Research with a Special Focus on Digital Medicine, Clinic for Gynaecology and Obstetrics University Clinic Düsseldorf, CIO Düsseldorf, Heinrich‐Heine‐University Düsseldorf Düsseldorf Germany

**Keywords:** active surveillance, Overtreatment, patient‐reported outcomes, prostate cancer, radical treatment

## Abstract

**Objective:**

To prospectively evaluate functional patient‐reported outcomes (PROs) in patients with low‐risk prostate cancer (PCa) managed with active surveillance (AS), nerve‐sparing radical prostatectomy (NS‐RP), non‐NS‐RP, or radiotherapy (RT).

**Patients and Methods:**

This multicentre prospective cohort study used data from the Prostate Cancer Outcomes (PCO) study in Germany, Austria and Switzerland, including 6265 patients with low‐risk PCa enrolled between 2016 and 2023. PROs were assessed at baseline and 12 months after treatment/enrolment using the 26‐item Expanded Prostate Cancer Index Composite Short Form (EPIC‐26), measuring urinary continence, bowel, sexual, hormonal, and irritative/obstructive symptoms. Mean score changes were compared with minimal important differences (MIDs) to determine clinical significance.

**Results:**

In all, 475, 4352, 813, and 625 patients received AS, NS‐RP, non‐NS‐RP, and RT, respectively. At 12 months, AS was associated with stable function across all EPIC‐26 domains. In contrast, both RP groups experienced significant declines in urinary continence (NS‐RP: −18 points; non‐NS‐RP: −26 points) and sexual function (NS‐RP: −35 points; non‐NS‐RP: −30 points), exceeding MID thresholds. Urinary continence did not decline after RT but clinically relevant declines occurred in irritative/obstructive urinary (−5 points), bowel (−7 points), hormonal (−5 points), and sexual function (−12 points). Age‐stratified analysis showed clinically significant declines in urinary and sexual function after NS‐RP across all age groups, with the greatest loss in sexual function among younger patients and the most pronounced continence impairment in the 70–79 years age group. In contrast, functional outcomes under AS remained stable in all age cohorts.

**Conclusion:**

Active surveillance is underutilised in the observed cohort. Prospective PCO data demonstrates that AS preserves urinary continence and sexual function compared to active treatment, supporting its role as the first‐line strategy for suitable candidates. Despite advancements including NS techniques, RP, and to a lesser extent RT, remain associated with substantial functional impairment even in younger men.

**Abbreviations**
AS: active surveillance
cT: clinical T stage
DKG: Deutsche Krebsgesellschaft (German Cancer Society)
DRKS: German Clinical Trials Register
EPIC‐26: 26‐item Expanded Prostate Cancer Index Composite Short Form
IQR: interquartile range
MID: minimally important difference
NeuroSAFE: neurovascular Structure‐Adjacent Frozen‐section Examination
NS: nerve sparing
PCa: prostate cancer
PCO: Prostate Cancer Outcomes (study)
PIVOT: Prostate cancer Intervention Versus Observation Trial
PRO(M): patient‐reported outcome (measure)
RP: radical prostatectomy
RT: radiotherapy
t0: baseline
t1: 12 months after the start of treatment or enrolment in AS

## Introduction

Prostate cancer (PCa) is the most commonly diagnosed and second leading cause of cancer death in men [1]. Despite a reduction in the mortality rate due to the early detection of aggressive tumours since the introduction of PSA screening, the early detection and radical treatment of indolent low‐risk tumours has led to extensive, avoidable morbidity and considerable costs due to overtreatment [2]. Compared to active surveillance (AS), local radical therapy leads to a significant deterioration in urinary, bowel and sexual function [3], and quality of life due to significant impairment of continence and potency in patients with low‐risk PCa [4]. AS aims to avoid or at least delay the potential complications and unwanted side effects of treatment while maintaining the chance of cure by detecting early signs of disease progression [5]. Thus, AS is recommended as the preferred treatment for low‐risk PCa in major clinical guidelines [6, 7, 8, 9]. Although AS rates have risen nationally, they remain suboptimal and vary widely among practices and clinicians [10, 11, 12].

Comparing the effectiveness and harms of radiotherapy (RT), radical prostatectomy (RP), and AS is critical for shared decision‐making [13]. The ongoing Prostate Cancer Outcomes (PCO) Study was established to monitor and improve treatment outcomes in men with PCa [14, 15]. It systematically collects and reports patient‐reported outcome measures (PROMs) using the validated 26‐item Expanded Prostate Cancer Index Composite Short Form (EPIC‐26) before and after treatment [16, 17]. The aim of our study was to evaluate the impact of AS compared to nerve‐sparing RP (NS‐RP), non‐NS‐RP, and RT on functional outcomes in patients with localised low‐risk PCa. For this we focused on treatment‐related side effects, including urinary incontinence, urinary irritative/obstructive symptoms, sexual dysfunction, bowel symptoms, and hormonal symptoms/vitality. The dataset includes patients treated in participating PCa centres located in Germany, Austria and Switzerland, certified by the German Cancer Society (Deutsche Krebsgesellschaft [DKG]) with baseline PROMs as well as follow‐up data after 12 months, with patient inclusion starting in 2016.

## Patients and Methods

### Data Collection

Certified PCa centres in Germany, Austria and Switzerland have recruited patients for the PCO study since 2016. Before definitive treatment or AS, patients with clinically localised PCa were requested to complete the EPIC‐26 questionnaire along with additional sociodemographic questions after providing informed consent at baseline (t0). Patients are asked to complete the EPIC‐26 questionnaire again 12 months after the start of treatment or enrolment in the case of AS (t1).

The PCO is an ongoing study and forms part of the True North (TrueNTH) Registry launched by the Movember Foundation (Melbourne, Australia). The Ethics Committee of the Medical Association of Berlin (Eth‐2/16) and local review boards have approved the study.

The analysis presented here includes data of patients with low‐risk PCa (PSA level <10 ng/mL, Gleason 6, clinical T stage [c]T1c–cT2a), classified according to the D’Amico risk classification, taking part in the PCO study who underwent any kind of definitive treatment or AS as standard of care strategy. The last patient recruited in the present study was in June 2023. Inclusion criteria for this analysis: low‐risk PCa, t0 and t1 questionnaire completed.

### Outcomes

The EPIC‐26 domain scores at 12 months after respective therapies (t1) were used as outcome measurements. The EPIC‐26 is a well‐established, PCa‐specific PRO questionnaire recommended by the International Consortium for Health Outcomes Measurement (ICHOM) that summarises responses in five domains: urinary incontinence, irritative/obstructive symptoms, bowel function, sexual function, and vitality/hormonal function [18]. The validated German translation of the EPIC‐26 was used [17]. All EPIC‐26 domain scores range between 0 and 100, with 0 indicating the poorest function. They are calculated using a scoring manual [19].

To compare the outcome measurements after therapy modalities, the mean difference of each score between baseline (t0) and after 12 months of beginning of definitive therapy or AS (t1) were calculated and compared with the minimal important difference (MID) ranges for each score domain: MIDs are the smallest change in a treatment outcome—such as PROMs—the patient would identify as important. Skolarus et al. [20] determined MIDs for each EPIC‐26 domain.

The sexual function domain focuses on the quality and frequency of erections (MID 10–12 points). The urinary incontinence (MID 6 points) and urinary irritative/obstructive symptom (MID 5 points) domains ask questions about frequency; amount of urinary leakage; and dysuria, haematuria, and urinary frequency. The bowel function domain (MID 4 points) focuses on bowel frequency, urgency, bleeding, and pain. The hormonal domain (MID 4 points) assesses symptoms such as hot flashes, gynaecomastia, low energy/fatigue, and weight change.

To evaluate age differences in functional outcomes in patients undergoing AS and NS‐RP, the cohort was stratified into four age groups: <60, 60–69, 70–79, and >79 years. Changes in EPIC‐26 domain scores were compared across all age groups to identify clinically significant differences exceeding the MID.

### Statistical Analysis

Baseline characteristics are presented across treatment groups, frequencies and proportions were used to present the categorical variables, while median with interquartile range (IQR) were used to present continuous variables. The mean (SD) was measured for each domain score. The mean score differences for AS and each therapy modality were calculated and compared with the corresponding MID. For each EPIC‐26 domain, a worsening or improvement with an absolute difference greater than or equal to the corresponding MID was considered a clinically relevant change. The number of patients with a clinically relevant decline (thus ≥1 MID) is reported together with other descriptives (Tables 2 and 3 and Table S1). Stratified analysis includes age (Table 3 and Table S1), as well as comorbidities (Tables S3 and S4). Data from routine clinical care are reported, thus missing values were not imputed. However, a drop‐out analysis was performed (Table S2).

The EPIC‐26 domain scores were analysed as observed, but we acknowledge that ceiling effects may limit the measurable decline in these subscales. This was considered in the interpretation of results.

## Results

**Fig. 1:**
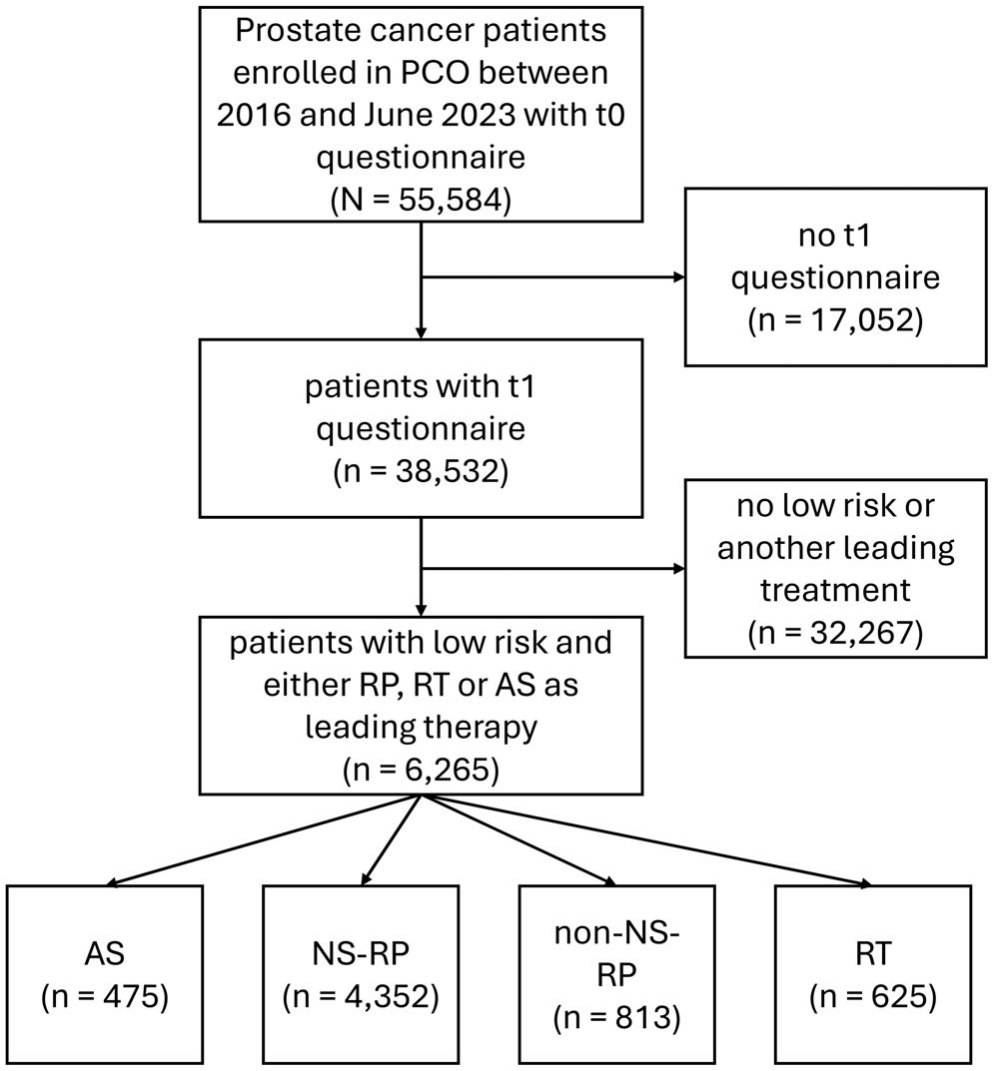
Flow chart for patient inclusion.

Between study initiation in 2016 and July 2024, a total of 55 584 patients were enrolled in the PCO study across 153 centres and completed the baseline (t0) questionnaire. Of these, 38 532 completed the 12‐month (t1) follow‐up questionnaire. Of these, 6265 patients had low‐risk PCa and were treated by either RP, RT or AS (with no combinations allowed except for AS before RT or RP) and were included in the final analysis (Fig. 1).

### Patient Characteristics

Table 1 shows the baseline patient characteristics. Of the 6265 patients enrolled in our study, 475 (7.5%) received AS, 4352 (69.5%) NS‐RP, 813 (13%) non‐NS‐RP, and 625 (10%) RT. The median age (IQR) for NS‐RP was 63 (58–68) years, which is slightly lower compared to 68 (62–73) years for AS, 68 (63–72) years for non‐NS‐RP, and 69 (63–75) years for RT. The majority of patients had cT1 disease, while cT2a was found in 11% in the AS, non‐NS‐RP and RT groups and slightly lower in the NS‐RP group at 8.9%. The initial median (IQR) PSA level was 4.90 (3.31–6.70) ng/mL in the AS group compared to 5.94 (4.76–7.43) in the NS‐RP, 6.20 (4.80–7.80) in the non‐NS‐RP, and 6.22 (4.83–7.74) in the RT groups. The majority of patients across all treatment groups reported no comorbidities (60–75%), with the highest proportion in the RT group (75%). Patients undergoing non‐NS‐RP had the highest rate of three or more comorbidities (2.5%), while this was least common among patients undergoing RT (0.5%).

**Table 1:** Patients’ characteristics.

Characteristic	AS, *N* = 475	NS‐RP, *N* = 4352	Non‐NS‐RP, *N* = 813	RT, *N* = 625
Age, years, median (IQR)	68 (62–73)	63 (58–68)	68 (63–72)	69 (63–75)
Comorbidities, *n* (%)				
Unknown	39 (8.2)	414 (9.5)	90 (11)	32 (5.1)
0	314 (66)	2874 (66)	489 (60)	470 (75)
1–2	113 (24)	1027 (24)	214 (26)	120 (19)
≥3	9 (1.9)	37 (0.9)	20 (2.5)	3 (0.5)
cT‐stage, *n* (%)				
T1	421 (89)	3963 (91)	720 (89)	556 (89)
T2a	54 (11)	389 (8.9)	93 (11)	69 (11)
T2b–c	0 (0)	0 (0)	0 (0)	0 (0)
T3	0 (0)	0 (0)	0 (0)	0 (0)
T4	0 (0)	0 (0)	0 (0)	0 (0)
cN stage, *n* (%)				
N0	475 (100)	4352 (100)	813 (100)	625 (100)
Gleason score, *n* (%)				
Gleason 6	475 (100)	4352 (100)	813 (100)	625 (100)
Gleason 7a	0 (0)	0 (0)	0 (0)	0 (0)
Gleason 7	0 (0)	0 (0)	0 (0)	0 (0)
Gleason 8	0 (0)	0 (0)	0 (0)	0 (0)
Gleason 9 or 10	0 (0)	0 (0)	0 (0)	0 (0)
Initial PSA level at diagnosis, ng/mL, median (IQR)	4.90 (3.31–6.70)	5.94 (4.76–7.43)	6.20 (4.80–7.80)	6.22 (4.83–7.74)
Insurance, *n* (%)				
Statutory	298 (74)	3016 (73)	638 (84)	491 (85)
Private	102 (25)	1069 (26)	124 (16)	86 (15)
None or other	5 (1.2)	27 (0.7)	2 (0.3)	2 (0.3)
Unknown	70	240	49	46
Education, *n* (%)				
Lower secondary school	97 (24)	1080 (26)	327 (43)	217 (38)
Entrance certificate for university	95 (24)	1222 (30)	135 (18)	103 (18)
Intermediate secondary school west	79 (20)	852 (21)	117 (15)	116 (20)
Entrance certificate for applied science	81 (20)	566 (14)	85 (11)	79 (14)
Intermediate secondary school east	34 (8.4)	300 (7.3)	76 (10)	46 (8.1)
Other	15 (3.7)	74 (1.8)	11 (1.5)	8 (1.4)
None	2 (0.5)	7 (0.2)	5 (0.7)	2 (0.4)
Unknown	72	251	57	54
Citizenship, *n* (%)				
German	312 (78)	3922 (96)	719 (95)	554 (96)
Other	87 (22)	169 (4.1)	39 (5.1)	21 (3.7)
Unknown	76	261	55	50

### Urinary Function

**Fig. 2:**
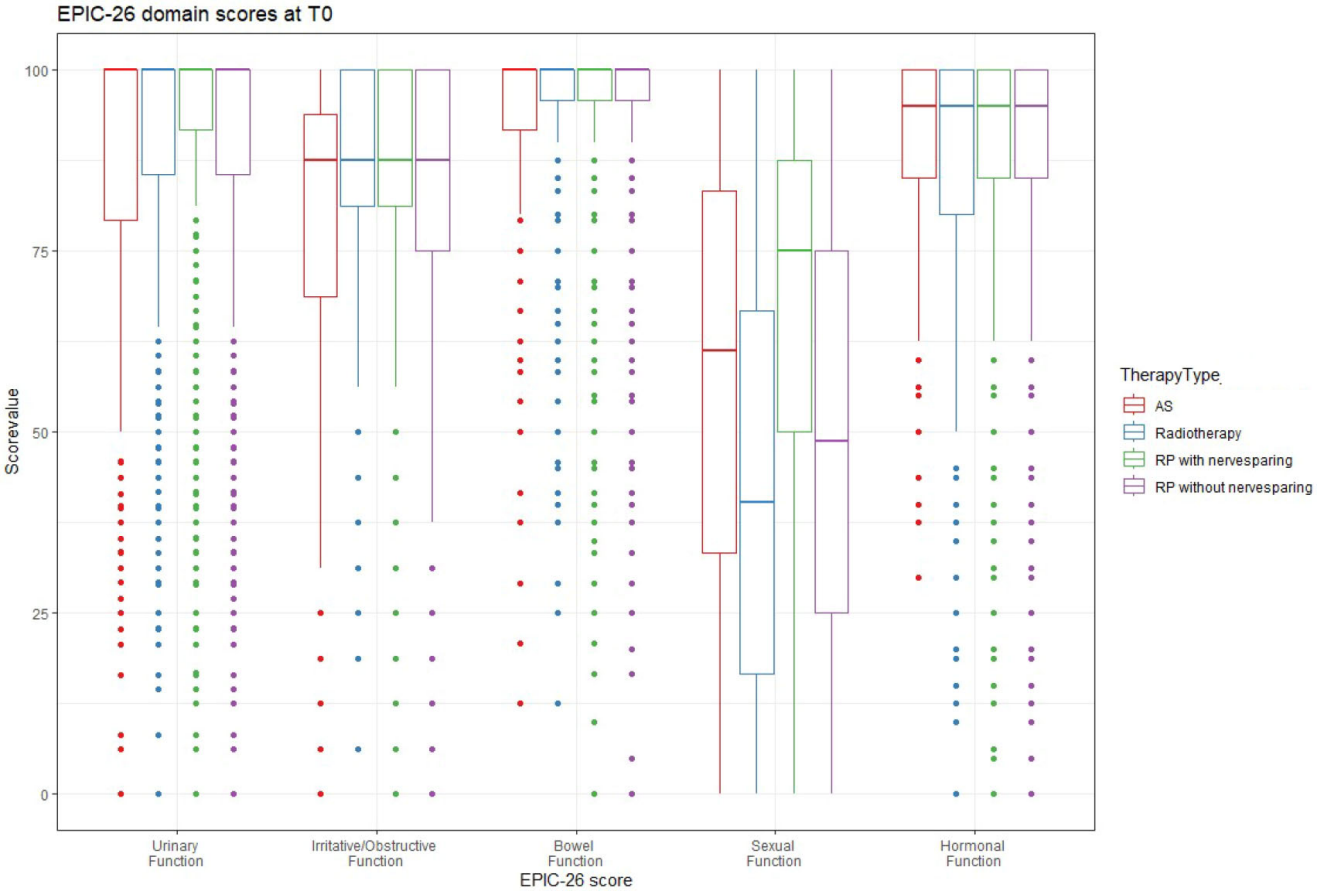
Box plot for EPIC‐26 domain scores at baseline (t0).

**Fig. 3:**
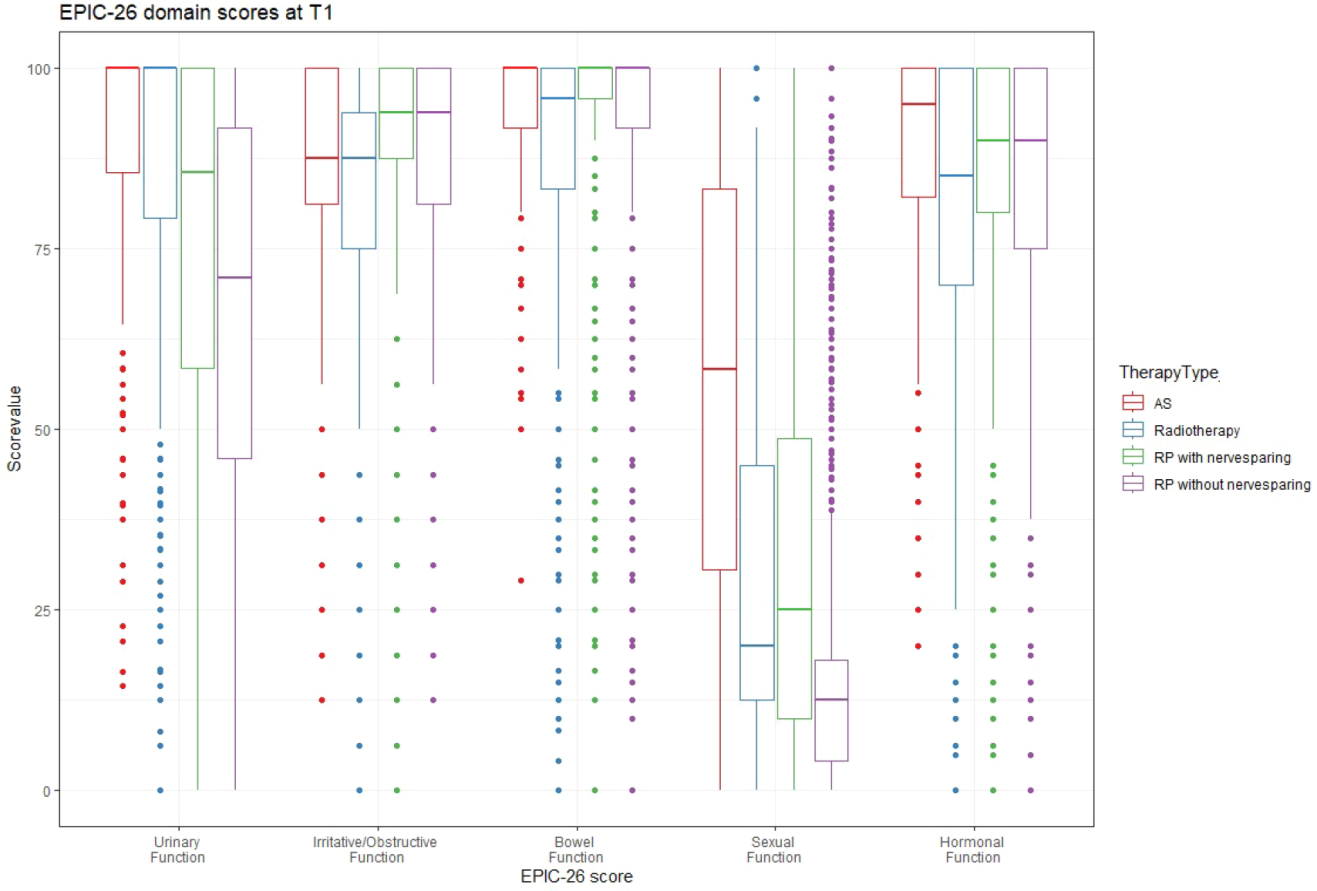
Box plot for EPIC‐26 domain scores 12 months after each therapy (t1).

Table 2 presents the mean EPIC‐26 scores for each domain before and 12 months after the different treatment groups. Each domain score before and after the different treatments is also graphically illustrated using box plots (Figs 2 and 3). The mean (SD) baseline urinary incontinence score was 87 (22) in the AS group, compared to 93 (13) in the NS‐RP group, 89 (17) in the non‐NS‐RP group, and 92 (14) in the RT group. While the AS and RT groups showed no changes in incontinence scores, the mean score worsened by more than four times the MID (−26 points) in the non‐NS‐RP group and three times the MID (−18 points) in the NS‐RP group at 12 months.

**Table 2:** The EPIC‐26 domain scores at baseline and 12 months after each therapy modality in patients with low‐risk PCa.

Characteristic	AS, *N* = 475	NS‐RP, *N* = 4352	Non‐NS‐RP, *N* = 813	RT, *N* = 625
Baseline urinary incontinence score, mean (SD)	87 (22)	93 (13)	89 (17)	92 (14)
Unknown, *n*	17	174	68	34
12‐month urinary incontinence score, mean (SD)	90 (16)	76 (26)	65 (29)	88 (19)
Unknown, *n*	22	103	32	24
Difference	+3	−17	−24	−4
Number of individuals with a deterioration ≥1 MID (% of those with t0 and t1 questionnaire)	101 (22.85)	2349 (57.47)	487 (67.73)	188 (32.98)
Baseline irritative/obstructive symptoms score, mean (SD)	78 (21)	86 (15)	83 (18)	88 (14)
Unknown, *n*	24	249	75	47
12‐month irritative/obstructive symptoms score, mean (SD)	86 (14)	91 (11)	88 (13)	83 (18)
Unknown, *n*	28	180	71	48
Difference	+8	+5	+5	−5
Number of individuals with a deterioration ≥1 MID (% of those with t0 and t1 questionnaire)	117 (27.21)	911 (22.97)	185 (27.05)	262 (48.34)
Baseline bowel function score, mean (SD)	94 (12)	96 (8)	95 (11)	96 (9)
Unknown, *n*	27	234	84	56
12‐month bowel function score, mean (SD)	94 (11)	95 (10)	93 (12)	89 (17)
Unknown, *n*	22	147	61	51
Difference	0	−1	−2	−7
Number of individuals with a deterioration ≥1 MID (% of those with t0 and t1 questionnaire)	92 (21.15)	971 (24.24)	204 (29.96)	245 (46.49)
Baseline sexual function score, mean (SD)	59 (29)	69 (26)	46 (28)	54 (29)
Unknown, *n*	23	129	36	32
12‐month sexual function score, mean (SD)	57 (30)	34 (27)	16 (15)	42 (28)
Unknown, *n*	13	78	25	20
Difference	−2	−35	−30	−12
Number of individuals with a deterioration ≥1 MID (% of those with t0 and t1 questionnaire)	111 (25.06)	3180 (76.52)	547 (72.07)	285 (48.97)
Baseline hormonal function score, mean (SD)	89 (14)	90 (14)	90 (14)	91 (14)
Unknown, *n*	21	194	57	41
12‐month hormonal function score, mean (SD)	89 (14)	87 (16)	85 (17)	86 (17)
Unknown, *n*	19	117	37	40
Difference	0	−3	−5	−5
Number of individuals with a deterioration ≥1 MID (% of those with t0 and t1 questionnaire)	162 (36.82)	1658 (40.74)	336 (46.09)	240 (43.45)

For irritative and obstructive symptoms, the mean (SD) baseline scores were 78 (21), 86 (15), 83 (18) and 88 (14) in the AS, NS‐RP, non‐NS‐RP and RT groups, respectively. After 12 months, no deteriorations were seen in the AS and both RP groups. In contrast, those undergoing RT experienced a worsening of their irritative and obstructive symptoms, with a decline equivalent to one MID (−5 points).

### Bowel Function

The baseline bowel function scores were nearly identical across all groups. After 12 months, only the RT group experienced a significant decline, exceeding 1.5 times the MID in the bowel domain score (−7 points). The other groups showed no changes in bowel function.

### Sexual and Hormonal Function

The mean baseline sexual function score was 69 in the NS‐RP group, while lower scores were observed in the AS (59), non‐NS‐RP (46), and RT (54) groups. After 12 months, stable sexual function was observed only in the AS group. In contrast, a substantial decline exceeding 3.5 times the MID was noted in the NS‐RP group (−35 points), while the non‐NS‐RP group experienced a deterioration of three times the MID (−30 points). The RT group showed a moderate decline just above the MID (−12 points).

The AS group maintained stable hormonal function with no changes observed. In contrast, the mean hormone domain score declined by one MID (−5 points) following RT and non‐NS‐RP. The decline after NS‐RP did not reach the MID threshold and was not clinically significant.

### Stratification of EPIC‐26 Domain Changes According to Age in the AS and NS‐RP Groups

To describe age differences on functional outcomes in patients undergoing AS, we stratified changes in EPIC‐26 domain scores by age groups. Across all age cohorts, urinary and sexual function remained stable over the follow‐up period (Table 3). A clinically significant deterioration was observed only in bowel function among the oldest patients.

**Table 3:** The EPIC‐26 scores in low‐risk patients at baseline and 12 months after treatment initiation under AS, stratified by age group.

Characteristic	Age, years			
	<60, *N* = 86	60–69, *N* = 194	70–79, *N* = 178	>79, *N* = 17
Baseline urinary incontinence score, mean (SD)	94 (11)	86 (23)	85 (23)	85 (28)
Unknown, *n*	2	4	8	3
12‐month urinary incontinence score, mean (SD)	93 (13)	90 (15)	88 (18)	82 (30)
Unknown, *n*	3	7	6	6
Difference	−1	+4	+3	−2
Number of individuals with a deterioration ≥1 MID (% of those with t0 and t1 questionnaire)	17 (20.99)	40 (21.62)	42 (25.45)	2 (18.18)
Baseline irritative/obstructive symptoms score, mean (SD)	79 (19)	78 (21)	77 (22)	82 (20)
Unknown, *n*	2	9	10	3
12‐month irritative/obstructive symptoms score	88 (15)	87 (13)	84 (15)	89 (13)
Unknown, *n*	4	7	11	6
Difference	+9	+9	+7	+7
Number of individuals with a deterioration ≥1 MID (% of those with t0 and t1 questionnaire)	22 (27.50)	51 (28.02)	43 (27.39)	1 (9.09)
Baseline bowel function score, mean (SD)	94 (13)	94 (12)	92 (13)	95 (7)
Unknown, *n*	2	9	14	2
12‐month bowel function score, mean (SD)	96 (8)	95 (9)	93 (13)	89 (12)
Unknown, *n*	3	6	9	4
Difference	+2	+1	+1	−6
Number of individuals with a deterioration ≥1 MID (% of those with t0 and t1 questionnaire)	19 (23.46)	34 (18.58)	32 (20.25)	7 (53.85)
Baseline sexual function score, mean (SD)	81 (19)	63 (27)	47 (27)	27 (19)
Unknown, *n*	2	7	13	1
12‐month sexual function score, mean (SD)	75 (26)	62 (26)	44 (28)	28 (23)
Unknown, *n*	1	2	7	3
Difference	−6	−1	−3	+1
Number of individuals with a deterioration ≥1 MID (% of those with t0 and t1 questionnaire)	22 (26.57)	45 (24.32)	43 (26.71)	1 (7.14)
Baseline hormonal function score, mean (SD)	89 (14)	90 (14)	89 (14)	88 (16)
Unknown, *n*	1	5	13	2
12‐month hormonal function score	88 (14)	90 (14)	88 (15)	89 (12)
Unknown, *n*	3	5	8	3
Difference	−1	0	−1	+1
Number of individuals with a deterioration ≥1 MID (% of those with t0 and t1 questionnaire)	28 (34.15)	70 (37.63)	57 (35.85)	7 (53.85)

After NS‐RP, we observed significant declines in both urinary incontinence and sexual function, with changes exceeding the MID across all age groups. Younger patients, who reported better baseline function in all domains, experienced more pronounced absolute decreases in sexual function. Specifically, those aged <60 years revealed declines as high as 3.5 times the MID (−37 points), while patients aged >79 years experienced a decline of 2.5 times the MID (−24 points). By examining urinary continence, the most considerable deterioration occurred in the 70–79 years age group, with reductions surpassing three times the MID (−20 points). Notably, younger patients (aged <60 years) also showed a significant decline of −16 points, which is >2.5 times the MID (Table S1). Bowel and hormonal function changes showed minimal non‐relevant changes. Irritative/obstructive function showed slight improvements across age groups.

## Discussion

Active surveillance is currently recommended in the major guidelines as the standard of care for patients with low‐risk PCa [7, 8, 9, 13]. Accordingly, recent studies have shown increasing adoption of AS in recent years [10, 21]. In a cohort study of 20 000 men treated in 350 urology practices in the United States, the rates of adoption of AS increased from 26.5% in 2014 to 59.6% in 2021 [10]. Despite this improvement, adoption remains suboptimal and shows significant regional variation, suggesting a further need for enhanced patient counselling to optimise AS rates [10, 22]. To facilitate informed treatment decision‐making, the potential benefits and risks of each management strategy must be discussed with patients, considering individual clinical and personal characteristics.

The existing evidence on the harms of radical PCa treatments comes mostly from selective study cohorts that may not reflect the real‐world population, including selected groups of patients with relatively younger age, better health status or better controlled disease stages compared to patients treated in routine care [23, 24, 25, 26, 27]. In addition, the literature is limited by the inconsistent measurement of functional outcomes, and many studies do not consider PROs or the role of confounding factors, e.g., age [28]. The current multicentre PCO study has many advantages, including real‐world data from patients with low‐risk PCa treated with AS in >140 PCa centres and various curative options, the prospective collection of PROs, and the large sample size of 6265 men with low‐risk disease alone.

Despite the guidelines’ recommendations and the increasing adoption of AS worldwide, RP in our study remains the most preferred treatment options for patients with low‐risk PCa, accounting for 82% of cases, compared to only 7.5% for AS. However, this observation may not accurately reflect the actual rates of adoption for various options. Although the PCO study mandates to include all consecutively treated patients, not all treated patients participated in the study. Patients undergoing AS are more frequently managed in the outpatient sector and are therefore less likely to be captured as centre‐based patients. As a result, this group is often underrepresented in PCO recruitment, which may partly explain the low proportion of AS cases in our dataset. The annual reports of the DKG‐certified cancer centres show a gradual, but still suboptimal increase in AS from 25% in 2016 to 36,8% in 2023 [11, 12]. Using MIDs, we provide a clear assessment of the clinical relevance and severity of changes in EPIC‐26 scores. RP was linked to the most significant deteriorations in urinary continence and sexual function. Moreover, the NS technique seemed not be sufficiently protective against functional deterioration. For urinary continence, a substantial decline was observed exceeding three (−18 points) and four (−26 points) times the MID in the NS‐RP and non‐NS‐RP groups, respectively. Similarly, a significant deterioration in sexual function was observed, exceeding 3.5 times (−35 points) the MID in the NS‐RP group and three times (−30 points) the MID in the non‐NS‐RP group. This could be attributed to the heterogeneity of NS techniques and the fact that thermal injury can cause reversible axonotmesis, a condition that may take up to 24 months for full recovery [29, 30]. Consequently, the 12‐month functional outcomes observed in the NS‐RP group in the present study may continue to improve over time. Notably, the recent multicentre, patient‐blinded, randomised controlled neurovascular Structure‐Adjacent Frozen‐section Examination (NeuroSAFE)PROOF study (ClinicalTrials.gov identifier: NCT03317990), including 344 patients with median follow‐up of 12 months, also demonstrated clear deterioration of sexual function in all patients regardless of NS guided by NeuroSAFE assessment [24]. Data from the prospective Comparative Effectiveness Analysis of Surgery and Radiation (CEASAR) trial observed a small but clear recovery from sexual function deterioration with longer follow up after RP [31].

Radiotherapy predominantly affected bowel and hormonal function resulting in a decline of >1.5 times the MID in the bowel domain score and just above the MID in the hormonal function domain. This is consistent with existing evidence suggesting RT is associated with higher rates of gastrointestinal morbidity [32, 33]. Ceiling effects in the bowel and hormonal domains, due to near‐maximal baseline scores, may have limited detectable change, and minimal observed differences should therefore be interpreted cautiously. The preservation of bowel function with other treatment options is still one of the significant advantages over RT. Our analysis of EPIC‐26 score changes suggests that AS is the management strategy most strongly correlated with the preservation of functional outcomes across all domains. Importantly, urinary and sexual functions remained preserved across all age cohorts undergoing AS. These findings support the suitability and tolerability of AS as the preferred management strategy [34]. The superiority of AS over the other curative options was also seen in other prospective studies that used EPIC scores [23, 35, 36, 37]. In the Utrecht Prostate Cohort, the EPIC questionnaire was evaluated at baseline and at various time points up to 12 months for the radical treatments and the AS groups. While all radical options showed significant deterioration in one or more domains at one or more follow‐up time points, AS remained stable across the different study time points [37]. In the multicentre Spanish study, AS was associated with the fewest side effects at 2 years compared to the other treatment options. The only side effect mentioned in relation to AS was sexual function, which worsened over the 2 years but remained better than with the other radical options [35]. This could be explained by the transition of 17% of patients from AS to active treatment [35]. Similar results were also reported in a larger cohort, but with a follow‐up of 1 year [36]. The sexual impairment in AS in these studies was not observed in our study, which showed stable sexual function at 12 months. However, the differences in the study cohorts limit the comparison between them. For example, in the latter Australian study comparing AS with radical treatment options, 76% of patients had intermediate‐ or high‐risk PCa and the AS rate was 15%, whereas in our study we only included low‐risk patients. Furthermore, psychosocial factors related to a cancer diagnosis and inconsistencies in the provision of sexual counselling and emotional support may contribute to the decline in sexual function observed in AS patients in these studies [38].

The superiority of AS over other treatment options was also seen in long‐term follow‐up studies using EPIC scores [23, 39]. An Australian study showed persistent clinically significant impairment of urinary continence and sexual function 15 years after RP irrespective of NS. In contrast, patients managed with AS showed more stable EPIC scores across all domains [39]. In addition, the randomised studies Prostate testing for cancer and Treatment (ProtecT) and Prostate cancer Intervention Versus Observation Trial (PIVOT) offer robust evidence supporting the mentioned findings. While low PCa‐specific mortality was seen after all treatment options, both trials found that RP led to significantly higher rates of urinary incontinence and erectile dysfunction compared to AS, with only a marginal survival benefit observed in the PIVOT trial [23, 25, 26]. Our findings complement results from both mentioned trials but extend them to a real‐world context. Those trials enrolled selected patients under randomised controlled conditions, whereas our cohort reflects unselected men treated in routine care. Both trials also included some intermediate‐ and high‐risk patients managed with AS, introducing potential tumour‐related influences on functional outcomes. In addition, their long follow‐up periods (15 and 19.5 years) allowed assessment of long‐term improvements after RP and delayed declines after RT, whereas our 12‐month follow‐up captures only early, real‐world outcomes. These differences in patient selection, disease risk, and follow‐up duration should be considered when comparing results across studies.

A major strength of our study is its large cohort of 6265 patients, which enabled the detailed observation of age‐specific changes in PROs across EPIC‐26 domains. Patients were divided into four age groups. Both younger and older patients experienced significant declines in urinary continence and sexual function after NS‐RP. Notably, younger patients (aged <60 years) had declines similar in magnitude to older patients in terms of urinary incontinence (−16 vs −20 points) and sexual function (−37 vs −31 points). These finding challenge the commonly held assumption that younger men are less affected by postoperative functional side effects [40]. Despite having better baseline function and similar absolute declines compared to those seen in older patients, their relative impact may be greater due to higher pre‐treatment expectations and activity levels. This suggests that age alone is not a protective factor against postoperative morbidity. In contrast, patients undergoing AS maintained stable urinary continence and sexual function across all age groups. Data from studies measuring decision regret after various treatment options of PCa support these findings. In a German multicentre study of 3408 patients, 10% reported a decision regret 15 years after RP [41]. In a prospective study of 2072 patients, regret was more common among low‐risk patients who underwent RP and RT than AS at 5 years follow‐up [42]. This highlights AS as the only viable, most quality‐of‐life‐preserving option for patients with low‐risk PCa, especially for those with longer life expectancy.

### Limitations

Several limitations must be considered when interpreting the results of this study. First, the non‐randomised design may introduce selection bias, as patients in the AS group were generally older and had lower PSA levels, which may potentially affect the outcomes. Second, although standardised comparisons using EPIC‐26 and MIDs were used, unmeasured confounders such as different surgical technique or RT protocols could influence results. Moreover, as the EPIC‐26 MIDs were derived from demographically distinct United States cohorts, their transferability to our German population may be limited. Thus, the MIDs should be considered indicative rather than definitive thresholds, acknowledging that cultural, structural, and baseline differences may influence the perception of functional changes. For that reason, a follow‐up project of the PCO study, the MID‐EPIC‐D study (German Clinical Trials Register [DRKS] identifier), is currently being conducted to estimate MIDs for the German‐speaking context. Third, the 12‐month follow‐up period may be too short to capture the full recovery, especially in the NS‐RP group. Nerve regeneration and recovery of sexual function may extend beyond the observed timeframe, potentially underestimating long‐term outcomes. Moreover, this period may also be insufficient to fully assess the side effects of RT, which often manifest later and may not yet be apparent within the first year. As previously mentioned, the MID‐EPIC‐D study will provide more insights thanks to a longer follow‐up period for PCO study participants. Fourth, the study did not assess psychosocial factors like mental health, decisional regret or patient satisfaction, which are important for treatment decision‐making. Fifth, the use of mean scores may mask the extent of adverse effects experienced by individual patients and limit the capacity to estimate individualised risk. Sixth, the small sample size in some AS subgroups could limit the detection of clinically relevant differences. Additionally, AS patients are often managed in the outpatient setting and are therefore underrepresented in PCO recruitment, which may introduce a selection bias. A substantial proportion of patients did not complete the 12‐month follow‐up questionnaire, which may introduce non‐response bias. Patients with particularly positive or negative experiences may have been less likely to participate in the follow‐up, potentially affecting the generalisability and accuracy of the reported outcomes. Finally, a high centre variability was already described elsewhere of the study group [15]. As our analysis is based on everyday clinical practice (‘real world’), we did not control this variability by using, i.e., hierarchical modelling.

## Conclusion

By utilising established assessment tools to evaluate each functional domain through the EPIC‐26 score and defining clinical significance using MIDs, this study provides real‐world evidence that facilitates decision‐making for patients with low‐risk PCa. The study is strengthened by its prospective and multicentre design. Additionally, unlike many previous studies, the inclusion of patients with comorbidities and non‐German participants enhances the generalisability of the findings. In summary, our study illustrates the diverse impacts of treatment modalities on functional outcomes in men with low‐risk localised PCa. AS has emerged as the only evidence‐based approach to preserving quality of life, and its adoption should continue to grow, especially in Germany.

## Author Contributions

Mulham Al‐Nader, Jan Fichtner, and Boris A. Hadaschik: conceived of the presented idea. Mulham Al‐Nader: manuscript writing, editing and submission. Claudia Kesch and Osama Mahmoud: participation in conception and design. Nora Tabea Sibert and Christoph Kowalski: protocol/project development, data analysis, Word supervision, manuscript revision and editing. Guenther Carl and Günter Feick: manuscript writing/editing. Martin Burchardt, Volker Zimmermanns, Lukas Prause, Bülent Polat, Andreas Blana, Marcus Horstmann, Matthias Saar, Petra Miglierini, Kinan Almansur, Lukas Hefermehl, Daniel Porres, Inga Peters, Kristina Wiens, Rein Jüri Palisaar, Alexander Winter, Andreas Neisius, Eva‐Maria Kunzmann, Nina Natascha Harke, Christian Bolenz, Mohamad Hatem Albarghouth, Lukas Manka, Mario Kramer, Ferdinand Luger, Thomas Knoll, Jesco Pfitzenmaier, Marko Brock, Julia Schittko, Jens Peter Sommer, Matthias Reichert, Sebastian Lenart, Philipp Huber, Sameh Hijazi, Anna Calderaro, Thomas Hermanns, and Jens Tonhauser: data collection and management, manuscript editing and revision. All authors read and approved the final version of the manuscript.

## Disclosure of Interests

Claudia Kesch has received consulting fees from Apogepha; research funding from Advanced Accelerator Applications (Novartis) and Curie Therapeutics; and travel support from Janssen Pharmaceuticals, Amgen, and Bayer, outside the submitted work. Osama Mahmoud has received a full scholarship from the Ministry of Higher Education of the Arab Republic of Egypt. Boris A. Hadaschik reports serving on advisory boards for Janssen, Bayer, ABX, Lightpoint, Amgen, MSD, Pfizer, and Novartis; speaker honoraria from Astellas and Janssen R&D; institutional royalties from Uromed; institutional research funding from AAA/Novartis, Bristol‐Myers Squibb, MS, and the German Research Foundation; an advisory role for German Cancer Aid; and leadership or speaker roles for DKG Association of Urological Oncology (AUO). Volker Zimmermanns is acting as a proctor, coaching other surgeons in robotic‐assisted surgery, mostly radical prostatectomy. Lukas Prause has received a European Association of Urology (EAU) European Urological Scholarship Programme (EUSP) fellowship grant (2024) and a fellowship grant from Stiftung Prostatakrebs Aarau (2025); consulting fees from Astellas and Bayer (to his institution); lecture honoraria from Bayer and Accord; and participation on a Data Safety Monitoring Board for Bayer and Astellas. Marcus Horstmann has received consulting fees from Johnson & Johnson; honoraria for lectures/presentations from Astellas and Johnson & Johnson; and support to attend meetings from Merck. Matthias Saar has obtained institutional grants from Novartis and Johnson & Johnson; consulting fees from Bayer AG, Johnson & Johnson, and Novartis; honoraria for lectures/presentations from those companies; and support for attending meetings from Bayer AG and Johnson & Johnson. Daniel Porres has received honoraria for presentations (Johnson & Johnson, Bayer), support for meeting attendance (Johnson & Johnson, Bayer), and participated on an advisory board for Bayer. Alexander Winter participated on a Data Safety Monitoring Board for Bayer and Novartis. Eva‐Maria Kunzmann has received support for attending meetings from MSD and Intuitive. Nina Natascha Harke has received payments or honoraria for lectures, presentations or educational events from Intuitive Surgical; support for attending meetings and travel from Bayer, Astellas, and Intuitive Surgical. She is Chairperson of the German Society of Urology (DGU) working group on laparoscopy and robot‐assisted surgery, Board member of the EAU Robotic Urology Section (ERUS), and on the ERUS Editorial Board of *European Urology Surgery in Motion*. Christian Bolenz has received honoraria from AstraZeneca, Johnson & Johnson, Bayer, Cepheid, and ERBE Elektromedizin GmbH; payment for expert reports as a court‐appointed medical expert in litigation cases; support for attending meetings from Johnson & Johnson; participation on Data Safety Monitoring Boards for Bristol‐Myers Squibb, Ferring Pharmaceuticals, and AstraZeneca. He is a member of the executive board of the DGU and of the steering committee for the S3 guideline groups for prostate cancer and bladder cancer. Matthias Reichert has received support for attending meetings from Boston Scientific. Sebastian Lenart has received honoraria for presentations from Janssen. Thomas Hermanns has received honoraria for presentations from Johnson & Johnson, Astellas, and Debiopharm; and participated on a Data Monitoring Board for Bayer. Christoph Kowalski and Nora Tabea Sibert are employees of the DKG. Mulham Al‐Nader, Jan Fichtner, Guenther Carl, Günter Feick, Martin Burchardt, Bülent Polat, Andreas Blana, Petra Miglierini, Kinan Almansur, Lukas Hefermehl, Inga Peters, Kristina Wiens, Rein Jüri Palisaar, Mohamad Hatem Albarghouth, Lukas Manka, Andreas Neisius, Mario Kramer, Marko Brock, Mario Kramer, Ferdinand Luger, Thomas Knoll, Jesco Pfitzenmaier, Julia Schittko, Jens Peter Sommer, Philipp Huber, Sameh Hijazi, Anna Calderaro, Jens Tonhauserhereby declare that they have no potential conflicts of interest.

## Funding Statement

Funding was received from the Movember Foundation through Help for Prostate Cancer Patients, the collaborating partner of the Movember Foundation in Germany. The analysis and interpretations are those of the authors and not of the Movember Foundation. The funders had no role in study design, data collection and analysis, decision to publish, or preparation of the manuscript.

## Supporting information


**Table S1** The EPIC‐26 scores in low‐risk patients at baseline and 12 months after NS‐RP, stratified by age group.
**Table S2** Analysis of baseline characteristics of dropouts (*n* = 2256).
**Table S3** Stratified comorbidities, only AS.
**Table S4** Stratified comorbidities, only NS‐RP.

## Data Availability

The data are that support the findings of this study are not publicly available due to containing information that could compromise the privacy of research participants but are available from the employees of the DKG Christoph Kowalski and Nora Tabea Sibert upon reasonable request.
