# Supplementary material for: Patient‐reported outcomes: active surveillance vs radical therapies in low‐risk prostate cancer
Source: BJU Int. 2026 Feb 16;137(4):716–27. doi: 10.1111/bju.70167 (PMC12962847; doi:10.1111/bju.70167)
Supplement: Supplementary file 1 — Table S1 The EPIC‐26 scores in low‐risk patients at baseline and 12 months after NS‐RP, stratified by age group. Table S2 Analysis of baseline characteristics of dropouts (n = 2256). Table S3 Stratified comorbidities, only AS. Table S4 Stratified comorbidities, only NS‐RP. [file BJU-137-716-s001.docx]

**Supplementary Table S1: EPIC-26 Scores in Low-Risk Patients at Baseline and 12 Months After NS-RP, Stratified by Age Group**

| **Characteristic** | **younger 60**  N = 1,363^1^ | **60 - 69**  N = 2,234^1^ | **70 - 79**  N = 747^1^ | **older 79**  N = 8^1^ |
| --- | --- | --- | --- | --- |
| Baseline urinary incontinence score | 96 (11) | 92 (14) | 91 (15) | 89 (15) |
| Unknown (n) | 30 | 96 | 47 | 1 |
| 12 months urinary incontinence score | 80 (24) | 76 (26) | 71 (28) | 82 (33) |
| Unknown (n) | 27 | 44 | 31 | 1 |
| Difference | -16 | -16 | -20 | -7 |
| Number of persons with a change >= 1 MID^2^ (% of those with t0 and t1 questionnaire) | 734 (56.16) | 191 (56.63) | 421 (62.74) | 3 (50.00) |
| Baseline irritative/obstructive symptoms score | 87 (15) | 85 (15) | 85 (16) | 80 (15) |
| Unknown (n) | 40 | 133 | 73 | 3 |
| 12 months irritative/obstructive symptoms score | 92 (11) | 91 (11) | 90 (12) | 91 (8) |
| Unknown (n) | 29 | 86 | 64 | 1 |
| Difference | +5 | +6 | +5 | +11 |
| Number of persons with a deterioration >= 1 MID (% of those with t0 and t1 questionnaire) | 335 (25.83) | 431 (21.21) | 143 (22.63) | 2 (40.00) |
| Baseline bowel function score | 97 (8) | 96 (9) | 97 (8) | 97 (5) |
| Unknown (n) | 36 | 113 | 83 | 2 |
| 12 months bowel function score | 95 (10) | 96 (10) | 95 (10) | 90 (9) |
| Unknown (n) | 21 | 82 | 44 | 0 |
| Difference | -2 | 0 | -2 | -7 |
| Number of persons with a deterioration >= 1 MID (% of those with t0 and t1 questionnaire) | 344 (26.34) | 467 (22.73) | 157 (24.61) | 3 (50.00) |
| Baseline sexual function score | 80 (22) | 66 (26) | 55 (27) | 38 (12) |
| Unknown (n) | 25 | 74 | 28 | 2 |
| 12 months sexual function score | 43 (29) | 32 (26) | 24 (22) | 14 (12) |
| Unknown (n) | 12 | 35 | 30 | 1 |
| Difference | -37 | -36 | -31 | -24 |
| Number of persons with a deterioration >= 1 MID (% of those with t0 and t1 questionnaire) | 1042 (78.58) | 1636 (76.77) | 497 (71.72) | 5 (83.33) |
| Baseline hormonal function score | 89 (14) | 90 (14) | 93 (12) | 96 (7) |
| Unknown (n) | 38 | 105 | 49 | 2 |
| 12 months hormonal function score | 84 (18) | 87 (16) | 90 (14) | 94 (8) |
| Unknown (n) | 22 | 58 | 37 | 0 |
| Difference | -5 | -3 | -3 | -2 |
| Number of persons with a deterioration >= 1 MID (% of those with t0 and t1 questionnaire) | 611 (46.86) | 798 (38.20) | 247 (36.81) | 2 (33.33) |
| ^1^Mean (SD)  ^2^Minimally important difference | | | | |

**Supplementary table S2: Analysis of baseline characteristics of drop outs (n = 2.256)**

| **Characteristic** | **AS**  N = 166^1^ | **Radiotherapy**  N = 145^1^ | **RP with nervesparing**  N = 1,646^1^ | **RP without nervesparing**  N = 299^1^ |
| --- | --- | --- | --- | --- |
| Age | 67 (61, 73) | 70 (66, 75) | 62 (58, 67) | 67 (62, 71) |
| Comorbidities |  |  |  |  |
| unknown | 14(8.4%) | 10 (6.9%) | 101 (6.1%) | 43 (14%) |
| none | 124(75%) | 111 (77%) | 1,165 (71%) | 179 (60%) |
| 1 - 2 | 27 (16%) | 20 (14%) | 370 (22%) | 65 (22%) |
| 3 or more | 1 (0.6%) | 4 (2.8%) | 10 (0.6%) | 12 (4.0%) |
| cT-stage |  |  |  |  |
| T1 | 142(86%) | 123 (85%) | 1,491 (91%) | 263 (88%) |
| T2a | 24 (14%) | 22 (15%) | 155 (9.4%) | 36 (12%) |
| T2b-c | 0 (0%) | 0 (0%) | 0 (0%) | 0 (0%) |
| T3 | 0 (0%) | 0 (0%) | 0 (0%) | 0 (0%) |
| T4 | 0 (0%) | 0 (0%) | 0 (0%) | 0 (0%) |
| cN Stage |  |  |  |  |
| N0 | 166(100%) | 145 (100%) | 1,646 (100%) | 299 (100%) |
| Gleason Score |  |  |  |  |
| Gleason 6  Gleason 7a  Gleason 7b  Gleason 8  Gleason 9 or 10 | 166(100%)  0 (0%)  0 (0%)  0 (0%)  0 (0%) | 145 (100%)  0 (0%)  0 (0%)  0 (0%)  0 (0%) | 1,646 (100%)  0 (0%)  0 (0%)  0 (0%)  0 (0%) | 299 (100%)  0 (0%)  0 (0%)  0 (0%)  0 (0%) |
| Initial PSA level at diagnosis (ng/mL) | 5.31 (4.00, 6.90) | 6.06 (4.43, 7.90) | 5.92 (4.68, 7.46) | 6.18 (4.69, 7.80) |
| Insurance |  |  |  |  |
| Statuatory | 106 (77%) | 114 (81%) | 1,158 (75%) | 243 (88%) |
| Private | 28 (20%) | 26 (19%) | 373 (24%) | 30 (11%) |
| None or Other | 3 (2.2%) | 0 (0%) | 18 (1.2%) | 3 (1.1%) |
| Unknown | 29 | 5 | 97 | 23 |
| Education |  |  |  |  |
| lower sedondary school | 41 (30%) | 49 (36%) | 362 (23%) | 114 (42%) |
| entrance certificat for university | 37 (27%) | 24 (18%) | 461 (30%) | 38 (14%) |
| intermediate secondary school west | 19 (14%) | 23 (17%) | 318 (21%) | 54 (20%) |
| entrance certificat for applied science | 20 (14%) | 22 (16%) | 213 (14%) | 25 (9.1%) |
| intermediate secondary school east | 17 (12%) | 14 (10%) | 145 (9.4%) | 38 (14%) |
| Other | 3 (2.2%) | 1 (0.7%) | 38 (2.5%) | 1 (0.4%) |
| None | 1 (0.7%) | 4 (2.9%) | 10 (0.6%) | 4 (1.5%) |
| Unknown | 28 | 8 | 99 | 25 |
| Citizenship |  |  |  |  |
| German | 109 (81%) | 130 (94%) | 1,439 (93%) | 263 (94%) |
| Other | 26 (19%) | 8 (5.8%) | 104 (6.7%) | 16 (5.7%) |
| Unknown | 31 | 7 | 103 | 20 |

| ^1^Median (Q1, Q3); n (%)  Abbreviations: AS, Active surveillance; RP, Radical Prostatectomy; PSA, prostate-specific antigen. |
| --- |

**Supplementary Table S3: stratified comorbidities, only AS**

| **Characteristic** | **unknown**  N = 39^1^ | **none**  N = 314^1^ | **1 - 2**  N = 113^1^ | **3 or more**  N = 9^1^ |
| --- | --- | --- | --- | --- |
| Baseline Urinary Incontinence score | 85 (25) | 90 (17) | 81 (28) | 67 (35) |
| Unknown (n) | 1 | 8 | 7 | 1 |
| 12 months Urinary incontinence score | 87 (21) | 91 (14) | 87 (19) | 72 (36) |
| Unknown (n) | 1 | 11 | 8 | 2 |
| Baseline irritative/obstructive symptoms score | 81 (21) | 80 (19) | 74 (24) | 60 (27) |
| Unknown (n) | 1 | 13 | 9 | 1 |
| 12 months Irritative/Obstructive symptoms score | 85 (18) | 86 (14) | 86 (16) | 87 (17) |
| Unknown (n) | 0 | 17 | 9 | 2 |
| Baseline bowel function score | 92 (12) | 94 (12) | 92 (12) | 96 (9) |
| Unknown (n) | 1 | 14 | 10 | 2 |
| 12 months Bowel function score | 91 (14) | 95 (9) | 93 (13) | 99 (2) |
| Unknown (n) | 1 | 12 | 7 | 2 |
| Baseline sexual function score | 60 (32) | 61 (28) | 53 (30) | 52 (36) |
| Unknown (n) | 0 | 16 | 7 | 0 |
| 12 months sexual function score | 55 (33) | 59 (28) | 51 (31) | 53 (31) |
| Unknown (n) | 0 | 10 | 3 | 0 |
| Baseline hormonal function score | 86 (17) | 91 (12) | 87 (15) | 75 (23) |
| Unknown (n) | 1 | 15 | 4 | 1 |
| 12 months hormonal function score | 88 (16) | 90 (12) | 85 (18) | 91 (9) |
| Unknown (n) | 0 | 12 | 5 | 2 |
| ^1^Mean (SD) | | | | |

**Supplementary table S4: stratified comorbidities, only NS-RP**

| **Characteristic** | **unknown**  N = 414^1^ | **none**  N = 2,874^1^ | **1 - 2**  N = 1,027^1^ | **3 or more**  N = 37^1^ |
| --- | --- | --- | --- | --- |
| Baseline Urinary Incontinence score | 92 (14) | 93 (13) | 93 (13) | 96 (10) |
| Unknown (n) | 22 | 103 | 46 | 3 |
| 12 months Urinary incontinence score | 72 (28) | 77 (26) | 77 (25) | 72 (28) |
| Unknown (n) | 4 | 68 | 29 | 2 |
| Baseline irritative/obstructive symptoms score | 84 (16) | 86 (15) | 85 (15) | 88 (12) |
| Unknown (n) | 38 | 150 | 57 | 4 |
| 12 months Irritative/Obstructive symptoms score | 90 (13) | 92 (11) | 91 (11) | 91 (12) |
| Unknown (n) | 20 | 107 | 51 | 2 |
| Baseline bowel function score | 96 (10) | 97 (8) | 97 (8) | 97 (7) |
| Unknown (n) | 37 | 141 | 54 | 2 |
| 12 months Bowel function score | 94 (11) | 96 (9) | 95 (10) | 97 (7) |
| Unknown (n) | 20 | 89 | 35 | 3 |
| Baseline sexual function score | 68 (26) | 70 (26) | 65 (26) | 63 (29) |
| Unknown (n) | 14 | 86 | 27 | 2 |
| 12 months sexual function score | 30 (26) | 36 (28) | 32 (26) | 26 (30) |
| Unknown (n) | 8 | 50 | 19 | 1 |
| Baseline hormonal function score | 90 (16) | 91 (14) | 89 (14) | 94 (11) |
| Unknown (n) | 29 | 121 | 40 | 4 |
| 12 months hormonal function score | 86 (17) | 87 (16) | 86 (17) | 88 (14) |
| Unknown (n) | 22 | 59 | 33 | 3 |
| ^1^Mean (SD) | | | | |
